# Sensitivity of *w*Mel and *w*AlbB *Wolbachia* infections in *Aedes aegypti* Puducherry (Indian) strains to heat stress during larval development

**DOI:** 10.1186/s13071-022-05345-0

**Published:** 2022-06-21

**Authors:** Kasinathan Gunasekaran, Candasamy Sadanandane, Devaraju Panneer, Ashwani Kumar, Manju Rahi, Sundaram Dinesh, Balakrishnan Vijayakumar, Muthuraman Krishnaraja, Sarala K. Subbarao, Purushothaman Jambulingam

**Affiliations:** 1 Medical Complex, Indian Council of Medical Research-Vector Control Research Centre (ICMR-VCRC), Indira Nagar, Puducherry, 605006 India; 2 Indian Council of Medical Research, Ramalingaswami Bhawan, Ansari Nagar, New Delhi, 110029 India

**Keywords:** *Aedes aegypti*, *Wolbachia*, Heat stress, Temperature tolerance, Dengue, India

## Abstract

**Background:**

ICMR-Vector Control Research Centre, Puducherry, India, developed two colonies of *Aedes aegypti* infected with *w*Mel and *w*AlbB *Wolbacia* strains called *Ae. aegypti* (Pud) lines for dengue control. The sensitivity of *w*Mel and *w*AlbB strains in *Ae. aegypti* (Pud) lines to heat stress was studied.

**Methods:**

*w*Mel and *w*AlbB infected and uninfected *Ae. aegypti* larvae (first to fourth instars) were reared in the laboratory to adults at 26 °C, 30 °C, 36 °C and 40 °C constant temperatures and also 26–30 °C, 26–36 °C and 26–40 °C diurnal cyclic temperatures. The adults were tested for *Wolbachia* infection. Experiments were also carried out rearing the larvae under simulated field conditions in summer (April and June) under sunlight using fully open and half open bowls and also under sunlight and natural shade.

**Results:**

At 36 °C and 40 °C constant temperatures, complete larval mortality was observed. At 30 °C and 26 °C, no larval mortality occurred, but *Wolbachia* density was relatively low in *w*Mel infected males compared to control (maintained at 26 ± 1 °C). At diurnal cyclic temperature of 26–40 °C, *Wolbachia* density was reduced in males of both the (Pud) lines, but not in females. At 26–36 °C, reduction in *Wolbachia* density was observed in *w*Mel males but not in *w*AlbB males. At 26–30 °C, no significant reduction in *Wolbachia* density was observed with *w*Mel and *w*AlbB strains. In simulated field conditions (April), under sunlight, the daytime water temperature reached a maximum of 35.7 °C in both full and half open bowls. No larval mortality occurred. *Wolbachia* frequency and density was reduced in *w*Mel-infected *Ae. aegypti* (Pud) males from both type of bowls and in females from full open bowls, and in *w*AlbB males from half open bowls. In June, rearing of larvae under sunlight, the first-instar larvae experienced a maximum daytime water temperature of > 38 °C that caused complete mortality. No larval mortality was observed in bowls kept under shade (< 32 °C).

**Conclusions:**

Exposure of larvae to higher rearing temperatures in the laboratory and simulated-field conditions reduced the densities of *w*Mel and *w*AlbB strains particularly in males, but the impact was more pronounced for *w*Mel strain. The actual effect of heat stress on the stability of these two *Wolbachia* strains needs to be tested under natural field conditions.

**Graphical Abstract:**

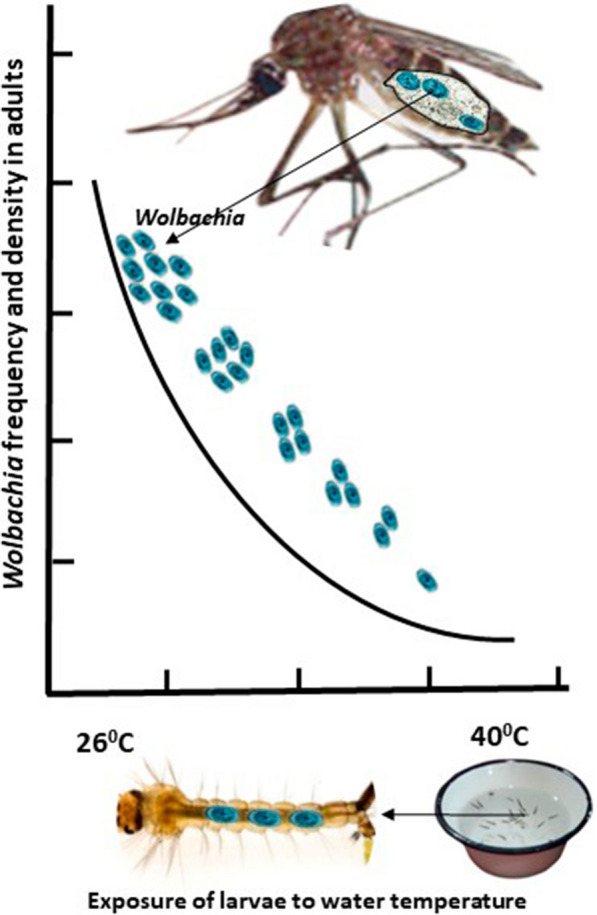

## Background

Dengue, a mosquito-borne, acute febrile illness, is a major public health problem in the tropics and the subtropics worldwide. According to the World Health Organization (WHO), over 129 countries are now endemic to dengue. It is estimated that 390 million dengue infections and 96 million dengue cases occur worldwide annually [1]. In India, outbreaks of dengue have been reported in 28 States and 6 Union Territories. A total of 687,890 dengue cases and 1110 deaths due to dengue infection were reported in the country during 2015–2020 [2]. Since there are no effective vaccines for community immunization and no drugs for treatment, control of the disease vector is the only option available for dengue control [3, 4]. *Aedes aegypti* is the major vector of dengue virus in India, and *Ae. albopictus* plays a secondary role in the transmission. Spraying of insecticides and larval source management are the measures carried out for vector control, but yield only a limited success [5–7]. This necessitated the development of alternative options for the control of dengue vector.

One such option is the use of *Wolbachia*-based strategy to prevent the transmission of dengue and other arboviral infections. *Wolbachia* is a genus of gram-negative intracellular bacteria under the order Rickettsiales and family Anaplasmataceae. These bacteria infect the invertebrate organisms and are naturally found in > 60% of the insects [8]. Transinfection of *Ae. aegypti* with *Wolbachia* strains, *w*AlbB [9, 10] and *w*Mel [11] has initially been shown to significantly reduce its vector competence, particularly to dengue virus under laboratory conditions. The World Mosquito Program (WMP) (formerly known as Eliminate Dengue Program), Monash University, Australia, has developed *Ae. aegypti* carrying *Wolbachia* strains (Australia), *w*Mel or *w*AlbB through embryonic microinjection. *w*Mel *Wolbachia* was isolated from *Drosophila melanogaster* [11] while *w*AlbB *Wolbachia* was from *Ae. albopictus* [12]. *Wolbachia,* a maternally transmitted endosymbiont, can spread to wild populations by inducing cytoplasmic incompatibility and interrupt disease transmission by interfering with virus replications [11, 13].

Currently, field release of *w*Mel-infected *Ae. aegypti* is underway in 11 countries to evaluate its effectiveness in controlling dengue [14], and in Kuala Lumpur, Malaysia, *w*AlbB-infected *Ae. aegypti* is successfully established among wild mosquito populations [15]. Successful invasion of *Wolbachia* into the native *Ae. aegypti* populations at the field sites of Australia, Brazil, Indonesia, Malaysia and Vietnam has been associated with varying levels of reduction in disease prevalence in the treated community. Recently, a cluster randomised trial in Yogyakarta city, Indonesia, demonstrated 77% reduction of virologically confirmed dengue cases post-release of *Wolbachia* mosquitoes [16]. Non-randomised controlled field trials in Indonesia [17] and Australia [18, 19] showed respectively 76% and 96% reduction of dengue incidence. In city-wide field trials, *Wolbachia* deployments caused 69% reduction of dengue cases in Brazil [20] and about 86% in Vinh Luong city, Vietnam [21].

To explore the alternate method of control of dengue transmitted by *Ae. aegypti*, Indian Council of Medical Research-Vector Control Research Centre (ICMR-VCRC), Puducherry, India, in collaboration with World Mosquito Program (WMP), Monash University, Australia, has successfully developed two new Indian *Wolbachia*-infected *Ae. aegypti* Puducherry (Pud) release lines through backcross experiments.

The newly developed Indian *Ae. aegypti* (Pud) release lines infected with *w*Mel or *w*AlbB *Wolbachia* strains are to be tested in field at a pilot scale to select a suitable strain for Indian conditions. Prior to field release, it is essential to assess the fitness of the release lines in the laboratory, as these mosquitoes should survive under field conditions for successful establishment of *Wolbachia* among the wild mosquito population. Besides, there are various environmental factors that would affect successful establishment of the inherited *Wolbachia* infections among the wild mosquito populations. Sensitivity to temperature is one such factor that could potentially limit the invasive capacity of a *Wolbachia* transinfected mosquito strain and also its ability to inhibit virus replication, thereby transmission.

Recent studies showed that *Wolbachia* strains *in Ae. aegypti* were vulnerable to higher temperatures [22–25]. Immature stages of *Ae. aegypti* grow in container habitats such as flower pots, water tanks, earthen pots, plastic barrels/drums, gutters, automobile tires, discarded utensils/containers, bottles, cans, scraps, etc., available in domestic and peri-domestic environments [26–28]. However, *Ae. aegypti* gravid females prefer to lay their eggs in shaded containers, the immature stages are also commonly found in containers that are fully exposed to sunlight [29, 30]. Ulrich et al. [22] reported that larval development of *Ae. aegypti* at higher water temperatures can experience attenuation in the *Wolbachia* levels. Exposure of larvae to high rearing temperature has been reported to reduce the ability of *Wolbachia* to induce cytoplasmic incompatibility and also the density of *Wolbachia* in adults [22, 25]. Therefore, in the current study, the ability of *w*Mel and *w*AlbB *Wolbachia* strains in *Ae. aegypti* (Pud) lines to tolerate higher temperatures was studied under laboratory and simulated-field conditions.

## Methods

### Mosquito strains and colony maintenance

Eggs of *w*Mel and *w*AlbB-infected *Ae. aegypti* Australian (Aus) strains were imported from WMP, Monash University, Australia, and reared at ICMR-VCRC, Puducherry, India. By backcrossing the females of *w*Mel or *w*AlbB-infected *Ae. aegypti* (Aus) strains with wild (field caught) *Ae. aegypti* Puducherry (Pud) males over six generations, two new release lines, viz., *w*Mel *Ae. aegypti* (Pud) and *w*AlbB *Ae. aegypti* (Pud), were developed and maintained for over 20 generations and at every generation, females of the release lines were outcrossed with 10% wild caught males. Eggs of uninfected wild *Ae. aegypti* (Pud) strain were collected using ovitraps from different sites of Puducherry, reared to adults, fed with human blood and allowed to oviposit. The F1 eggs were used for temperature sensitivity studies.

### Temperature sensitivity studies under laboratory conditions

The tolerance of *w*Mel and *w*AlbB infections to two temperature regimens was studied under laboratory conditions. In the first regimen, first-instar larvae of *w*Mel/*w*AlbB *Ae. aegypti* (Pud) release lines and uninfected wild *Ae. aegypti* (Pud) line were exposed to temperature maintained constantly at 30 °C, 36 °C and 40 °C up to pupal stage. In the second regimen, the larvae were reared at diurnal cyclic temperatures of 26–30 °C, 26–36 °C and 26–40 °C to pupae.

The eggs of the *w*Mel/*w*AlbB *Ae. aegypti* (Pud) release lines and the uninfected wild *Ae. aegypti* (Pud) line were hatched in cooled boiled (deoxygenated) water containing brewer’s yeast (0.2 g/l). Batches of 25 first-instar larvae of each line were released separately into 500-ml glass beakers containing 300 ml of tap water and the beakers were placed inside a water bath-stirred (14 l capacity, temperature range: 5–90 °C; EQUITRON Medica Pvt Ltd Mumbai, India) till the larvae pupated. The water baths were set to maintain temperature constantly at 30 °C or 36 °C or 40 °C or at daytime cycling temperatures of 26–30 °C, 26–36 °C and 26–40 °C. Four replicates (each with 25 larvae) were kept for each temperature regimen and for each line. Simultaneously, larvae of *w*Mel/*w*AlbB *Ae. aegypti* (Pud) and uninfected wild *Ae. aegypti* (Pud) were maintained constantly at 26 °C ± 1 °C outside a water bath as controls for each experiment. Larvae were fed with fish food, Tetramin tropical tablet @ 2.00 mg per larva, during the experiments. Water temperature inside the water bath and the glass beakers was recorded using submerging data loggers (Tiny tag aquatic, Gemini data loggers, UK). Five-day-old emerged adults from all the experiments were screened for *Wolbachia* frequency and density. The experiments were replicated twice using different batches of first-instar larvae.

### Temperature sensitivity studies under natural sunlight

Temperature sensitivity studies were also carried out under sunlight during summer months (April and June) at ICMR-VCRC premise, Pondicherry district, Union Territory of Puducherry. Pondicherry has a tropical climate with moderate variation of temperature and rainfall. Summer starts in April and lasts up to early June when maximum temperature may reach 41 °C (106 °F). The average maximum temperature ranged from 28 °C in January to 37 °C in May and the average minimum temperature fluctuated between 20 °C (January) and 27 °C (May). The average annual rainfall is about 1260 mm and almost 68% of it falls during October to December.

#### Experiment I—exposure to sunlight with full open/half open bowls

In this experiment, we used two types of plastic bowls (500 ml capacity; 14 cm diameter and 6.5 cm depth), fully open and half open (partially covered). The bowls were partially covered using chart sheet paper. Batches of 50 first-instar larvae of each line were separately released in to plastic bowls (fully/half open) containing 300 ml of tap water and placed under sunlight. Three replicates were maintained for each line and type of bowls. The bowls were covered with nylon net at sunset (18.00 h) to prevent the wild mosquitoes from ovipositing and the net covers were removed the next day morning.

#### Experiment II—exposure to direct sunlight and natural shade

In this experiment, batches of 50 first-instar larvae of *w*Mel/*w*AlbB *Ae. aegypti* (Pud) release lines and wild *Ae. aegypti* (Pud) line were released separately into plastic bowls (500-ml capacity) containing 300 ml of water and placed under sunlight. Three replicates (each with 50 larvae) were maintained for each line. Simultaneously, batches of 50 first-instar larvae of each line (in three replicates) were released separately into 500-ml plastic bowls with 300 ml of water and placed under tree shade. For both experiment I and II, larvae of *w*Mel/*w*AlbB *Ae. aegypti* (Pud) and uninfected wild *Ae. aegypti* (Pud) were maintained at a constant temperature of 26 °C ± 1 °C as control. Water temperature in the bowls was recorded at hourly intervals using submerging data loggers for the entire duration of the experiment.

In both experiments I and II, equal quantities (2.00 mg/larva) of larval food (crushed Tetramin tablets) were used to feed the larvae until their pupation. On day 5 and 6, pupae from each replicate were collected and returned to the insectary and placed inside labelled Bugdorm cages (15 × 15 × 15 cm) for emergence. The emerged adults were provided with 10% sucrose solution (soaked in cotton wool) and maintained at a constant temperature of 27 ± 2 °C and a relative humidity of 80 ± 10% up to day 5 post- emergence. Five-day-old, non-blood-fed adults (both males and females) of each line and temperature regimen were screened for *Wolbachia* frequency and density.

### Screening for *Wolbachia* frequency and density

The frequency and the density of *Wolbachia* infections in *Ae. aegypti* lines were estimated using real-time PCR. Individual mosquitoes were screened for the presence of *Wolbachia* by multiplex real-time Taqman PCR assay, using the primers and the probes targeting *WSP* gene for *w*Mel, Ankyrin repeat domain gene for *w*AlbB, respectively. Simultaneously, RPS gene (ribosomal protein), specific for *Ae. aegypti,* was used as positive control. Density of *Wolbachia* in individual mosquitoes was estimated using Comparative C_t_(^2−ΔΔCt^) method following the standard operating procedure (SOP) of WMP, Monash University, Australia, September 2018, on “Screening of *Wolbachia* (*w*Mel and *w*AlbB) in adult mosquitoes using triplex qPCR (96 well)” [31].

### Statistical analysis

Data were expressed as mean (SD) and range (minimum, maximum). Mann-Whitney U test was used to determine the difference in *Wolbachia* density between the experimental and control groups at different temperature regimens. Paired t-test was used to compare the temperatures between the full and half open bowls. *P*-value < 0.05 was considered statistically significant. All statistical analyses were done in statistical software STATA 14.2 version (College Station, TX, USA).

## Results

### Tolerance to constant temperatures in laboratory

On exposure to the constant temperature of 40 °C, complete mortality of larvae of both *w*Mel and *w*AlbB *Ae. aegypti* (Pud) release lines and also of the wild *Ae. aegypti* (Pud) was observed. Similarly, when exposed to 36 °C maintained constantly, all the larvae of *w*AlbB and wild (Pud) lines, except seven larvae of *w*Mel, died and on screening the adult mosquitoes emerged from those seven alive larvae (4 ♂ and 3 ♀); none were found positive for *Wolbachia*. On exposure to the temperature constantly maintained at 30 °C and also to controls (maintained at a constant temperature of 26 °C ± 1 °C), *Wolbachia* frequency was 100% in both males and females of *w*Mel and *w*AlbB *Ae. aegypti* (Pud) release lines.

The *Wolbachia* density (Table 1) in both males and females of *w*Mel and *w*AlbB *Ae. aegypti* (Pud) release lines exposed to the constant temperatures of 30 °C did not differ significantly from the corresponding controls (maintained at 26 ± 1 °C) (*w*Mel female: U = 25, Z = 0.74, P = 0.46; *w*AlbB male: U = 30, Z = 0.21, *P* = 0.83; *w*AlbB female: U = 15, Z = 1.79, *P* = 0.07 by Mann-Whitney U test), except in *w*Mel males (Pud), in which the density was significantly lower than the control (U = 5, Z = 2.84, *P* = 0.005) (Table 2).

**Table 1:** *Wolbachia* frequency and density in *w*Mel and *w*AlbB *Ae. aegypti* (Pud) release lines on exposure of their larvae (first instar) to constant temperature of 30°C compared to 26 ± 1°C (Control)

Strain	Temp (constant)	Replicate	No. of larvae exposed	No. emerged/ screened		*Wolbachia* frequency (%)		Average *Wolbachia* density (range)	
				**♂**	**♀**	**♂**	**♀**	**♂**	**♀**
*w*Mel (Pud)	30 °C	8	200	54	46	100	100	1.56 ± 0.39 (1.08–2.18)	10.16 ± 3.91 (5.59–17.05)
	26 ± 1°C Control	8	200	53	47	100	100	2.73 ± 0.77 (1.57–3.81)	11.61 ± 3.76 (7.54–17.75)
*w*AlbB (Pud)	30 °C	8	200	51	49	100	100	28.95 ± 5.28 (22.43–39.86)	22.99 ± 6.78 (15.68–34.64)
	26 ± 1°C Control	8	200	50	50	100	100	30.00 ± 7.51 (20.69–38.51)	29.52 ± 6.90 (16.39–39.46)

**Table 2:** *Wolbachia* density in *w*Mel and *w*AlbB *Ae. aegypti* (Pud) release lines on exposure in larval stage to temperatures maintained at constantly and different ranges of diurnal cyclic temperatures in laboratory and under sunlight (natural) compared to the respective line exposed to a constant temperature of 26 ± 1 °C (control) in laboratory

Temperature/condition	Experiment				Control (26 ± 1°C constant)			
	Replicate	*n*^#^	Mean (SD)	Min.–max.	Replicate	*n*^#^	Mean (SD)	Min.–max.
*w*Mel male								
26 °C to 40 °C*	8	200	1.33 (0.92)	0.39–2.41	8	200	9.17 (7.67)	2.05–19.14
26 °C to 36 °C*	8	200	2.20 (0.94)	1.14–3.91	8	200	3.07 (0.56)	2.42–3.93
26 °C to 30 °C	8	200	3.99 (0.89)	2.44–4.85	8	200	3.85 (1.82)	2.07–6.44
30 °C*	8	200	1.56 (0.39)	1.08–2.18	8	200	2.73 (0.77)	1.57–3.81
Full open bowls*	3	150	2.05 (0.54)	1.66–2.66	4	100	16.02 (3.47)	11.38–19.14
Half open bowls*	3	150	0.75 (0.24)	0.47–0.93	4	100	16.02 (3.47)	11.38–19.14
*w*Mel female								
26 °C to 40 °C	8	200	7.36 (4.48)	1.50–12.17	8	200	8.29 (3.67)	4.57–13.75
26 °C to 36 °C	8	200	6.20 (3.90)	2.55–11.76	8	200	7.29 (4.28)	3.19–12.44
26 °C to 30 °C	8	200	11.44 (5.40)	1.35–16.70	8	200	10.67 (4.08)	4.63–15.90
30 °C	8	200	10.16 (3.91)	5.59–17.05	8	200	11.61 (3.76)	7.54–17.75
Full open bowls*	3	150	10.91 (0.93)	9.89–11.72	4	100	5.05 (0.55)	4.57–5.80
Half open bowls	3	150	5.57 (4.16)	3.05–10.37	4	100	5.05 (0.55)	4.57–5.80
*w*AlbB male								
26 °C to 40 °C*	8	200	31.68 (6.03)	24.29–38.68	8	200	37.35 (4.21)	28.47–41.15
26 °C to 36 °C	8	200	29.67 (7.27)	17.39–36.23	8	200	23.96 (11.09)	13.65–40.43
26 °C to 30 °C	8	200	18.10 (14.61)	3.61–33.44	8	200	29.63 (2.88)	25.53–33.80
30 °C	8	200	28.95 (5.28)	22.43–39.86	8	200	30.00 (7.51)	20.69–38.51
Full open bowls	3	150	32.99 (4.81)	28.23–37.85	4	100	35.82 (5.70)	28.47–40.39
Half open bowls*	3	150	23.46 (0.91)	22.77–24.50	4	100	35.82 (5.70)	28.47–40.39
*w*AlbB female								
26 °C to 40 °C	8	200	24.09 (11.63)	12.23–39.69	8	200	31.06 (8.11)	20.87–43.07
26 °C to 36 °C	8	200	24.25 (10.01)	10.90–43.56	8	200	17.77 (11.14)	9.07–41.56
26 °C to 30 °C*	8	200	19.35 (4.46)	14.77–27.91	8	200	23.61 (2.93)	20.03–28.65
30 °C	8	200	22.99 (6.78)	15.68–34.64	8	200	29.52 (6.90)	16.39–39.46
Full open bowls	3	150	34.53 (5.15)	29.33–39.63	4	100	26.22 (5.19)	20.87–32.67
Half open bowls	3	150	23.65 (4.80)	20.25–29.15	4	100	26.22 (5.19)	20.87–32.67

^*^Statistically significant

^#^Number of larvae exposed

### Tolerance to diurnal cyclic temperatures in laboratory

The frequency of *Wolbachia* was 84.6–100% in both males and females of *w*Mel and *w*AlbB *Ae. aegypti* (Pud) release lines at the diurnal cyclic temperatures of 26–40 °C, 100% (except one replicate of *w*Mel female that showed a frequency of 92.3%) at 26–36 °C and also 100% (except one replicate of *w*Mel (Pud) females which had a frequency of 91.7%) at 26–30 °C.

**Fig. 1:**
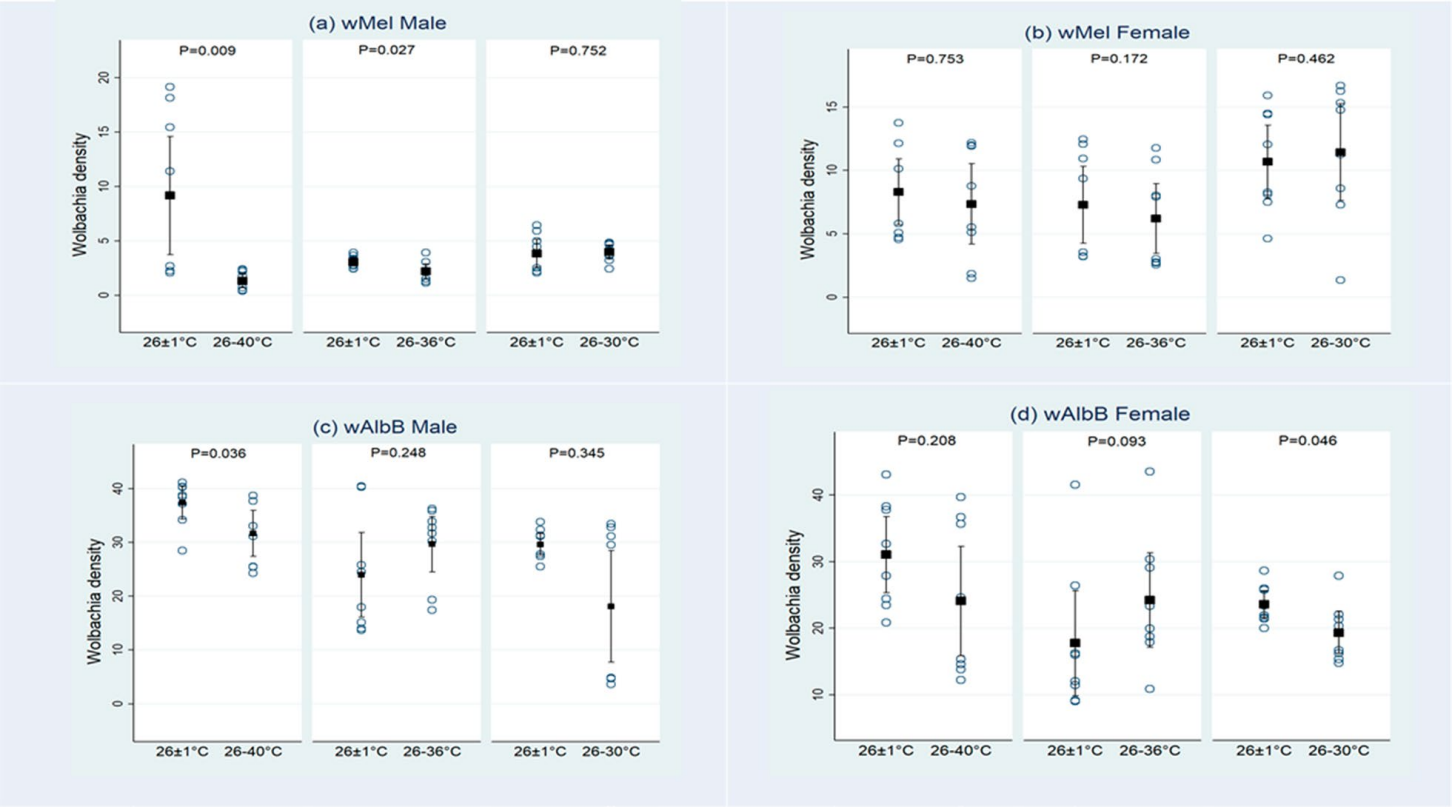
*Wolbachia* density in *w*Mel male (**a**) and female (**b**) and *w*AlbB male (**c**) and female (**d**) *Ae. aegypti* (Pud) lines on exposure of larvae to diurnal cyclic temperatures of 26–30 °C, 26–36 °C, 26–40 °C and control at 26 ± 1 °C. The dark square mark represents the mean density, and the upper and lower vertical lines represent 95% confidence limits

At the diurnal cyclic temperature of 26–40 °C, the *Wolbachia* density in *w*Mel (Pud) and *w*AlbB (Pud) males was significantly lower than the controls (*w*Mel male: U = 7, Z = 2.63, *P* = 0.009, *w*AlbB male: U = 12, Z = 2.1, *P* = 0.036), whereas in the females, the density was not significantly different from the controls (*w*Mel female: U = 29, Z = 0.32, *P *= 0.75; *w*AlbB female: U = 20, Z = 1.26, *P* = 0.21) (Fig. 1, Table 2). At 26–36 °C, in *w*AlbB (Pud) males (*w*AlbB male: U = 21, Z = 1.16, *P* = 0.25) and in females of both the release lines, the density did not differ significantly from the controls (*w*Mel female: U = 19, Z = 1.37, *P* = 0.17; *w*AlbB female: U = 16, Z = 1.68, *P* = 0.09). However, there was a significant reduction of the density in *w*Mel (Pud) males compared to the control (*w*Mel male: U = 11, Z = 2.2, *P* = 0.03) (Fig. 1, Table 2). At 26–30 °C, the density of *w*Mel in both males and females did not differ significantly from the control (*w*Mel male: U = 29, Z = 0.32, *P* = 0.75; *w*Mel female: U = 25, Z = 0.74, *P* = 0.46). While the density in *w*AlbB males was not significantly different from the control (U = 23, Z = 0.95, *P* = 0.34), the difference in the density between *w*AlbB (Pud) females and the corresponding control was at the statistical limit (*w*AlbB female: U = 13, Z = 2.00, *P* = 0.05) (Fig. 1, Table 2).

### Temperature tolerance on exposure to sunlight

Temperature tolerance was studied by exposing the larvae directly to direct sunlight. In the first regimen, two types of bowls, full and half open (partially covered), were deployed. The mean minimum water temperature was 26.7 ± 0.66 °C (range: 26.02–27.7 °C) in the full open bowls and 26.8 ± 0.67 °C (range: 26.12–27.8 °C) in the half open bowls kept under sunlight. The mean maximum water temperature was 38.54 ± 2.24 °C (range: 35.7–41.2 °C) and 38.1 ± 2.04 °C (range: 35.6–40.4 °C) in full and half open bowls, respectively. Overall and over time, the diurnal fluctuations of water temperature did not differ significantly between full and half open bowls (t_(5)_ = 0.422; *P* = 0.673, by paired samples t-test).

**Fig. 2:**
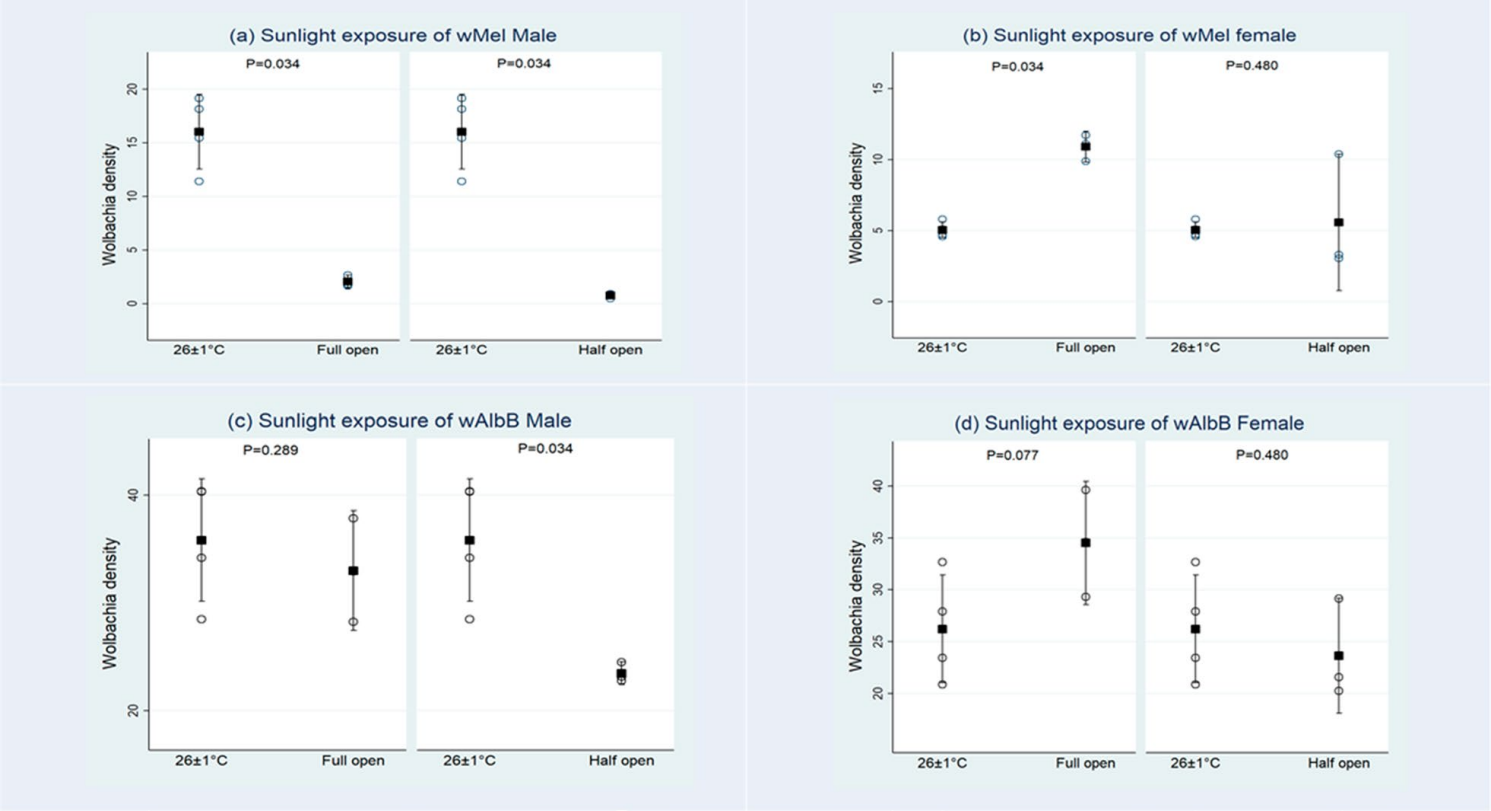
*Wolbachia* density in *w*Mel male (**a**) and female (**b**) and *w*AlbB male (**c**) and female (**d**) *Ae. aegypti* (Pud) lines on exposure of larvae in full and half open bowls to sunlight and control at 26 ± 1 °C. The dark square mark represents the mean density, and the upper and lower vertical lines represent 95% confidence limits

After the exposure of larvae to sunlight in full open bowls, the frequency of *w*Mel ranged from 68.2–85.0% in adult males and 82.4–91.6% in females. It was 100% in both males and females of *w*AlbB (Pud) *Ae. aegypti*. When larvae were reared in half open (partially covered) bowls under sunlight, the *Wolbachia* frequency ranged from 20.83 to 80.95% in *w*Mel males and 28.57–66.66% in females. It was 100% in *w*AlbB (Pud) males and females. The *Wolbachia* density in the two release lines and controls after exposure to sunlight in full and half open bowls is presented in Fig. 2 and Table 2. When compared to the control (maintained constantly at 26 °C ± 1 °C), there was a significant reduction of *Wolbachia* density in *w*Mel males in both types of bowls (full open: U = 0, Z = 2.12, *P* = 0.03; half open: U = 0, Z = 2.12, *P* = 0.03) and also in *w*AlbB males exposed in half open bowls (U = 0, Z = 2.12, *P* = 0.03) (Table 2). However, no significant reduction was observed in *w*AlbB female in both types of bowls (full open; U = 1, Z = 1.17, *P* = 0.08; half open: U = 4, Z = 0.71, *P* = 0.480 and also in *w*AlbB males in full open bowls (U = 3, Z = 1.06, *P* = 0.29).

### Temperature tolerance in sunlight vs shade

In this experiment, larvae were exposed to sunlight without any shade on the bowls and to full natural shade in June, the warmest month of the year. On day 1, the ambient temperature at 06.00 h was 27 °C and it reached a maximum of 41.8 °C at 12.00 h. From 12.00 to 14.00 h, the temperature was > 40 °C. The water temperature in the experimental bowls kept under sunlight was in the range of 38.2 to 39.3 °C at 12.00 h, and on day 1, complete mortality of first-instar larvae was observed in these bowls. In the bowls kept under shade, the water temperature reached a maximum of 30.6 °C during the daytime and no larval mortality was observed. The experiment was discontinued because of complete mortality of first-instar larvae in bowls kept under sunlight and the larvae kept under shade were also not reared to adults to screen the *Wolbachia* frequency and density.

## Discussion

In this study, we examined the sensitivity/tolerance of *w*Mel and *w*AlbB infections in *Ae. aegypti* (Pud) lines to heat stress under laboratory and simulated field conditions. Rearing of larvae (first to fourth instars) of *Wolbachia*-infected and -uninfected *Ae. aegypti* (Pud) at the constant temperatures of 36 °C and 40 °C resulted in complete/near complete mortality. At 30 °C constant temperature, there was no larval mortality, but reduction of *Wolbachia* density was observed in *w*Mel *Ae. aegypti* adult males. However, exposure of *Wolbachia*-infected and -uninfected *Ae. aegypti* larvae to constant rearing temperatures may not simulate/represent the actual field conditions; these experiments provided a critical thermal maximum (≥ 36 °C) beyond which mortality of *Ae. aegypti* larvae occurs.

At the diurnal cyclic temperature of 26–40 °C, *Wolbachia* density was reduced in males of both the release lines, but not in females, indicating that *Wolbachia* infection in males was sensitive to heat stress. Furthermore, the reduction of density was observed only in *w*Mel males but not in *w*AlbB males at 26–36 °C, which points out relatively more sensitivity of *w*Mel to heat stress. *Wolbachia* strains in *Ae. aegypti* have been reported to differ in their response to heat stress [23, 32]. Rearing of *w*Mel- and *w*Mel-Pop-CLA-infected *Ae. aegypti* (Aus strain) larvae at diurnal cyclic temperature of 26–37 °C reduced the density of *Wolbachia* in adults drastically; in contrast, *w*AlbB infection was maintained at high density [23]. Exposure of larvae to rearing temperature fluctuated between 27 °C and 37 °C reduced the density of *w*AlbA, *w*AlbB and *w*Mel; however, the impact was more pronounced for *w*Mel [32]. These findings were from the laboratory studies and it was not clear whether the effects of heat stress on *Wolbachia* are transient and will be restored back in the absence of heat stress. Foo et al. reported that *Wolbachia* density got partially recovered in female offspring of parents that experienced heat stress under laboratory conditions [24].

Experiments under simulated field conditions were carried out during summer (April and June). In the first experiment, two types of bowls, full and half open (partially covered), were used to rear larvae under natural sunlight. Half open bowls were deployed to provide partial shade to the larvae while rearing, expecting that the temperature of rearing water should be less compared to full open bowls. However, no significant difference in the daily fluctuations of rearing temperature was observed between the two types of bowls probably because of small size of the containers (500 ml capacity, with 300 ml of water) used for the experiment. Though the chart paper used to partially close the bowls provided shade, it might have also limited the dissipation of heat from the bowl water. During the experiment with full/half open bowls conducted in April, first-instar larvae were exposed to a maximum water temperature of 35.7 °C and second, third, and fourth instars and pupae to a maximum daytime water temperatures of 37.5 °C, 37.9 °C, 40.4 °C and 41.2 °C, respectively. No larval and pupal mortalities were observed. However, there was a reduction of *Wolbachia* frequency and density in *w*Mel-infected *Ae. aegypti* (Pud) males and females. For *w*AlbB infected *Ae. aegypti*, there was a reduction of density in *w*AlbB males and not in females, indicating *w*AlbB infection in females was less sensitive to heat stress. In experiment I (exposure in full/half open bowls), the maximum daytime water temperature in the bowls on day 1 was 35.7 °C, which did not kill any first-instar larvae. However, in experiment II (exposure to sunlight/shade) conducted in June, first-instar larvae experienced a maximum daytime water temperature that fluctuated between 38.2 °C and 39.3 °C in different replicates, which was on the higher side. This caused complete (100%) mortality indicating the critical thermal point and that first-instar larvae were most vulnerable to heat stress.

In the current study, reduction of *Wolbachia* density was observed at a high rearing temperature under laboratory as well as simulated field conditions and the results were consistent with the earlier observations [22, 23, 25, 32]. The thermal death point for *Wolbachia*-infected and -uninfected *Ae. aegypti* larvae was ≥ 36 °C under both laboratory and simulated field conditions. Comparison of densities of *w*AlbB and *w*Mel in *Ae. aegypti* (Pud) release lines showed *w*Mel was more sensitive to higher temperatures, while *w*AlbB was more resilient. Similarly, comparison of *Wolbachia* density between male and female mosquitoes indicated that infection in males was highly sensitive to diurnal cyclic temperatures, matching the observation by Ross et al. [23]. It has been reported that *w*AlbB strain has a better thermostability profile compared to *w*Mel in mosquito larvae and the strain has been selected for deployment in Kuala Lumpur, Malaysia, for dengue control [15]. However, the temperatures set in the laboratory experiments were meant to mimic larval habitat temperatures in the field, but did not truly represent those experienced by mosquitoes in field conditions [18].

For a successful field release strategy, *Wolbachia* infections should persist at high frequencies and block virus transmission under field conditions for many years following deployment [33]. Recent studies reported that *Wolbachia* strains are vulnerable to high temperatures [22, 23, 25, 32]. *Aedes aegypti* larvae are commonly found in container habitats in the peri-domestic environment, often experiencing wide diurnal fluctuations of temperature, especially in habitats that are exposed to sunlight. The effectiveness of the strategy could therefore be influenced by environmental temperature, which may decrease *Wolbachia* frequency and density, thereby reducing the ability of *Wolbachia* to invade and persist in the population and block virus replication. Despite being sensitive to heat stress, *w*Mel strain has been released successfully in several tropical countries where high temperatures may have a deleterious effect on *Wolbachia*. In large-scale city-wide field releases, spatial and seasonal heterogeneity in *w*Mel invasion was observed. In a quasi-experimental trial in Nitero'i, Brazil, deployments of *w*Mel-infected *Ae. aegypti* mosquitoes during 2017–2019 resulted in heterogeneous invasion and spread of *w*Mel in to the local *Ae*. *aegypti* populations at an infection frequency of 33–90% by March 2020 [34]. The landscape of Nitero'i is more vulnerable to temperature variations and the exposure of immature *Ae*. *aegypti* to very high temperatures in small water containers has been attributed as one of the environmental factors leading to slower and heterogeneous *w*Mel invasion. In Rio de Janeiro, Brazil, *w*Mel-infected adults were released into two residential areas between August 2017 and March 2020. At the end of the monitoring period, the *w*Mel invasion and spread to the local *Ae*. *aegypti* populations was found to be heterogeneous, and the overall infection rate was 50–70% in the first site and 30–60% in the other site [35]. Releases of *w*Mel *Ae. aegypti* into two small communities in Nha Trang City in central Vietnam resulted in a seasonal heterogeneity of *w*Mel invasion and spread into the local *Ae*. *aegypti* populations with a reduced prevalence of *Wolbachia* infection in mosquitoes during the hot dry season, followed by an increased prevalence during the cooler season, and such seasonal variation in *Wolbachia* infection prevalence in mosquitoes was associated with elevated temperature and was possibly due to imperfect maternal transmission of *Wolbachia* [36]. These studies suggested that the maternal transmission of the two *Wolbachia* strains can become unstable in *Ae. aegypti* at high temperatures and is likely to tend to recover back with optimum temperature conditions. Hence, it is important to better understand various factors affecting invasion dynamics of the *Wolbachia* strains in different settings and seasons to optimise the release strategies.

Long-term studies showed that despite its susceptibility to heat stress, *w*Mel strain has established and persisted in the field at a high frequency within the *Ae. aegypti* population in many locations in Cairns, Australia, and dengue transmission declined to zero in the release areas [37]. It has been reported that *w*Mel infection has remained stable so far in terms of virus blockage [13] and its effects on fitness [38]. Cairns, Australia, has a tropical climate. The average annual maximum temperature was 29 °C with 62% humidity. During summer, the average temperature ranged from 23.6 °C to 31.4 °C. On rare occasions, the daytime temperature in summer reached 36 °C to 40 °C. In a recent field study in Australia, Ross et al. [39] reported that heat stress on *w*Mel infection had only temporary effects on *Wolbachia* frequency and density once the infection had been established in nature. In November 2018, Cairns, Australia, experienced a heatwave of 43.6 °C; subsequently, a sharp decline in the frequency and density of *Wolbachia* was observed in the field population of *Ae. aegypti*, but recovered back closer to 100% 4 months later.

The climate of India comprises a wide range of weather conditions across a vast geographic scale and topography. There are seven climatic regions in India starting from tropical desert to mountain Climate. In most parts of the country, temperature tends to exceed 40 °C during summer months (April–June). Data on water temperature in various types of larval habitats prevalent in these regions during summer are not available, although observations in the simulated studies indicate there could be a difference (lower) of 1–3 °C from the ambient temperature. Considering the climatic conditions in various parts of India, field releases of *Ae. aegypti* mosquitoes transinfected with *Wolbachia* strains should be undertaken during the seasons except the summer months, i.e., from April to June, so that the *Wolbachia* strains will become established among the wild population without undergoing any heat stress.

## Conclusions

The success of *Wolbachia* release programs depends on the stability of *Wolbachia* strains in nature. Monitoring directly under natural conditions is important to assess the effects of heat stress on *Wolbachia* strains. Therefore, pilot field releases need to be undertaken to generate evidence on the stability of the *w*Mel- and *w*AlbB-infected *Ae. aegypti* (Pud) lines and their thermal tolerance/sensitivity and finally to select a suitable strain for field release in Indian conditions.

## Data Availability

Supporting data for the conclusion of this article are included within the article. The raw data used for and analysed during this study are available upon reasonable request.

## Abbreviations

Pud: Puducherry
Aus: Australia
WMP: World Mosquito Program
